# Prediction of heavy metal ion distribution and Pb and Zn ion concentrations in the tailing pond area

**DOI:** 10.1371/journal.pone.0308916

**Published:** 2024-09-26

**Authors:** Pengfei Wu, Bowen Chen, Runzhi Li, Ruochen Li

**Affiliations:** 1 School of Civil Engineering, Liaoning Technical University, Fuxin, Liaoning, China; 2 School of Mechanics and Engineering, Liaoning Technical University, Fuxin, Liaoning, China; 3 China Coal Technology and Engineering Group Shenyang Research Institute, Shenyang, Liaoning, China; 4 Triumph Science & Technology Co., Ltd, Bengbu, Anhui, China; University of Peshawar National Centre of Excellence in Geology, PAKISTAN

## Abstract

The pollution caused by tailings ponds has resulted in ecological damage, with soil contamination significantly impacting the daily lives of residents in the vicinity of mining areas and the future development of mining areas. This study assesses the transport status of heavy metal pollution in tailings areas and predicts its impact on future pollution levels. This study focused on lead–zinc tailing ponds, exploring the spatial and chemical distribution characteristics of heavy metals based on the distributions of Pb, Zn, As, Cu, Cr, Cd, Hg, and Ge ions. The concentrations of the major heavy metal ions Pb and Zn in tailings ponds were predicted via the exponential smoothing method. ① The total accumulation of Pb and Zn in the mine tailings ranges from 936.74~1212.61 mg/kg and 1611.85~2191.47 mg/kg, much greater than the total accumulation of the remaining six heavy metals. The total accumulation of associated heavy metal Cu was high, and the lowest total heavy metals were Hg and Ge at only 0.19 mg/kg and 1.05 mg/kg. ② The analyses of soil heavy metal chemical forms reveal that the heavy metals Pb and Zn had the highest exchangeable state content and state ratio and the strongest transport activity in the industrial plaza and village soils. Pb and Zn are the heavy metals with the greatest eco-environmental impacts in the mining area. ③ The predicted results show that the soil concentrations of the heavy metals Pb and Zn around the tailings area in 2026 are 1.335 and 1.191 times the predicted time starting values. The concentrations of the heavy metals Pb and Zn at the starting point of the forecast are already 3.34 and 3.02 times the upper limits of the environmental standard (according to environmental standards for gravelly grey calcium soils). These results have significant implications for heavy metal pollution risk management.

## Introduction

The environmental pollution and hazards of tailings pond areas have attracted widespread attention in the industry. Due to improper disposal practices in tailings pond areas, not only are the health and safety of residents in the surrounding areas affected [1,2] but serious damage is inflicted upon the soil ecosystem of the mining area. When the concentration of heavy metals in tailings is high, heavy metals can leach, infiltrate, and percolate into groundwater [3,4]. The circulation of groundwater systems [5] can affect the environmental quality of surrounding areas [6]. Additionally, agricultural and sideline products contaminated by heavy metals can directly endanger human health [7,8]. Therefore, accurate assessment and prediction of heavy metal pollution in tailings pond areas are of significant scientific and practical importance for pollution risk management and soil remediation.

Regardless of whether tailings pond areas are located in forested or cultivated lands, various toxic heavy metals, including Pb, Zn, As, Cu, Cr, Cd, Hg, and Ge, are inevitably introduced [9–11]. Over time, these harmful heavy metal ions can cause serious pollution to the surrounding soil and aquatic environments [12]. Various heavy metal ions contained in tailings pond areas exist in the soil through adsorption and deposition mechanisms [13]. An environmental pollution risk assessment can be used to effectively analyse heavy metal ions in sediments from mining areas. The risk level of heavy metal ions in sediments can be assessed based on different carriers (such as animals, plants, and humans) [14]. However, heavy metal ions can migrate between different species, indicating that eliminating heavy metal ions from soil in mining areas is extremely challenging [15].

In recent decades, many investigators have conducted in-depth research on soil heavy metal pollution caused by mining areas, with dissolution mechanisms identified as the primary cause of Pb and Zn release [16]. A decrease in pH enhances the leaching ability of Pb, with the release of metal elements initially occurring rapidly and then reaching a near-stable state [17]. Furthermore, metal-enriched zones gradually expand over time [18], causing harm to organisms through bioaccumulation in the food chain [19–21]. The spatial distribution of heavy metals in tailings is related to the geochemical characteristics of the tailings but not to the depth of the tailings profile. Additionally, the content of heavy metals decreases with increasing distance from the mining area [22]. Several researchers have investigated the hydraulic conductivity sequence of tailings: coarse tailings > fine tailings > medium tailings > fine tailings [23]. Regarding the migration and leaching process of heavy metals, Tabelin et al. calibrated the groundwater/AMD flow and solute transport in a two-dimensional reactive transport model [24]. The leaching process of metal ions in tailings is controlled by internal and external diffusion or surface chemical reactions [25,26]. To minimize the waste of resources, Liu et al. reduced the resources used for large-scale sampling by making extensive predictions in key areas [27]. However, traditional methods of direct measurement of heavy metal content in soil suffer from drawbacks such as uneven and uncontrollable sampling points and difficulty in predicting future trends [28]. Accurately determining the spatial distribution of heavy metal ions in tailings ponds and their surrounding environment and predicting their concentrations are crucial. Predicting and assessing heavy metal content not only provides direction for the development of mining areas but also offers important scientific evidence and decision support for soil environmental pollution. This is conducive to the sustainable development of mining areas [29,30].

In summary, numerous studies have assessed pollution levels based on the current state of contamination, while only a few have been able to predict and establish predictive models for tailings pollution by analysing the spatial and chemical distributions of heavy metals in tailings. This study focused on Pb‒Zn tailing ponds, exploring the spatial and chemical distribution characteristics of heavy metals based on the distributions of Pb, Zn, As, Cu, Cr, Cd, Hg, and Ge ions. The exponential smoothing method was used to predict the concentrations of the main heavy metal ions, Pb and Zn, in tailings ponds. This study had three objectives: (1) Analyse the changes in heavy metal content in tailings around Pb/Zn tailings ponds. (2) Evaluate the heavy metal pollution status in the surrounding soil of lead-zinc tailings ponds. (3) Investigate the spatial and chemical distribution characteristics of heavy metals. (4) Establish predictive models to predict soil heavy metal pollution around tailings ponds. These results can provide a basis for studying the migration law, pollution mode, and mechanism of heavy metal pollutants in tailings ponds and offer necessary technical support for the treatment of heavy metal pollutants in lead-zinc mining areas and the establishment of tailings pond impermeability technology systems.

## Materials and methods

### Sampling of tailings and surrounding soil

**Fig 1:**
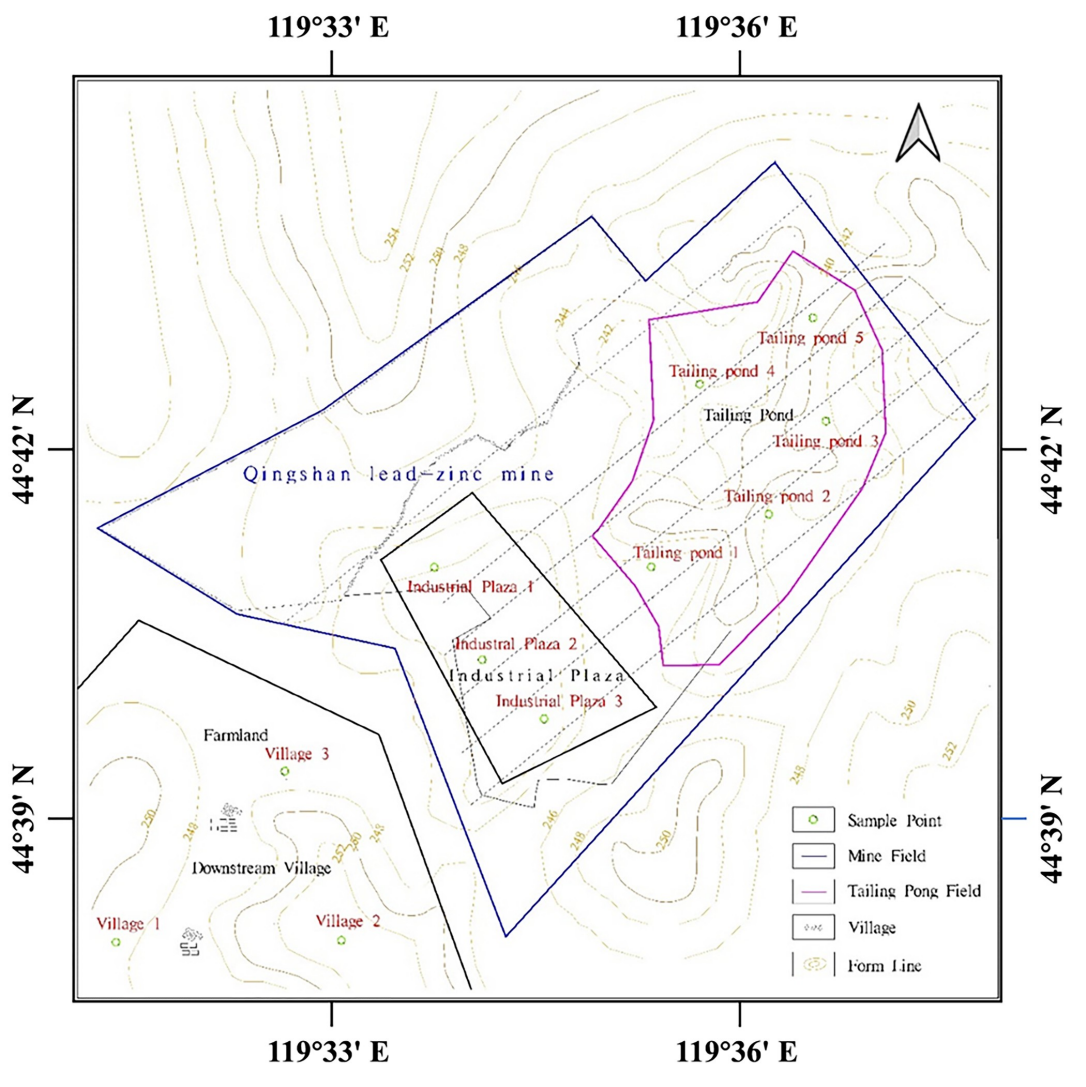
Distribution of sampling points in the tailing pond of the Qingshan lead-zinc mine.

Based on the fundamental principles of representativeness, uniformity, and comparability of sampling points, our team selected sampling locations near pollution sources to ensure the reliability and repeatability of outdoor sampling. This approach ensures the acquisition of representative samples for subsequent analysis and research. From the tailings discharge port to the tailings dam of the Qingshan tailings pond, three sampling points were set up at 100 m intervals. A soil auger was used for sampling, as shown in Fig 1, with a sampling depth of 2 m and a tailing sand sample collected every 10 cm. To prevent cross-contamination during sample collection and processing and to further enhance the rigor of sampling, the tailings samples were mixed into sealed polyethylene plastic bags, brought back to the laboratory, allowed to dry naturally, and sieved through 100 mesh.

Soil samples were collected from the industrial plaza of the lead-zinc mine and the surrounding villages. Two sample collection points were randomly selected, each sampling point was approximately 5 m long. Three sample points were selected, 0–20 cm surface soil samples were collected, and the environmental conditions and plant types at the sampling points were recorded in detail [31]. All soil samples were kept in sealed plastic sealed bags and numbered sequentially. Debris was removed from the samples, then the samples were naturally air-dried, sieved through 100 mesh and then bottled.

### Sample heavy metal detection

(1) Determination of the mineral phases and physicochemical properties of tailings and soil samples

The soil mineral phase distribution was determined using an X-ray diffractometer (D/MAX-2600pc). The microstructural features of the specimens were captured using a JSM-7900 F Scanning Electron Microscope (SEM) with an accelerating voltage of 15.0 kV. The soil particle composition was determined using a Mastersizer 2000. Soil organic matter (OM) was measured using the low-temperature external thermal potassium dichromate oxidation method. The sample pH was determined by the pH meter method (PHS-3C) using a soil-liquid ratio of 1:2.5 for proportioning.

(2) Heavy metal detection in tailings and soil samples

In the laboratory, rubber gloves and disposable PE protective suits were worn, and operations strictly conducted in accordance with regulations. Total heavy metals were dissolved by wet digestion with an electric hot plate, i.e., the specimen samples were dissolved using a high-temperature decoction with HCl-HNO_3_-HF-HClO_4_. The digestion solution is based on the standard Q/GD001-2002 of the National Geological Experimental Test Centre, which is a closed dissolution sample of trace elements in rocks, soils and aqueous sediments. The instrument analysis is specified in Table 1.

**Table 1:** Heavy metal detection methods and instruments.

Element	Analytical methods	Check out the limit value (mg/L)	Identification criteria (mg/L)	Testing equipment
Pb	Atomic absorption Spectrophotometer	17.23	5	Atomic absorption photometer (ShimatsuAA-6600, Japan)
Zn		154.80	100	
Cu		4.45	100	
Cr		0.6	1	
Cd		0.18	0.3	
Hg	Atomic fluorescence Spectrophotometer	Not detected	0.1	Flame atomic fluorescence photometer (HaiGuang AFS-3999, Beijing)
As		1.30	5	

Heavy metal morphology analysis using the BCR sequential extraction method [32]. BCR three-step extraction was used to determine the exchangeable state (weak acid extraction state), easily reduced state (Fe/Mn oxide-bound state), easily oxidized state (organic and sulphide-bound state) and residual state of the four heavy metals. The method categorizes the changes in natural and artificial environmental conditions into four patterns. At the same time, the selective extractant is fully applied in a weak to strong manner so that the miscibility effect is minimized. All the samples were analysed in 3 replicates to eliminate human error. According to Table 1, the maximum leaching toxicity of the heavy metal ions Pb and Zn in the tailings exceeded the standard, and the concentration was higher than the identification standards for hazardous waste.

## Results and analysis

### Basic characteristics of the lead-zinc tailings

**Fig 2:**
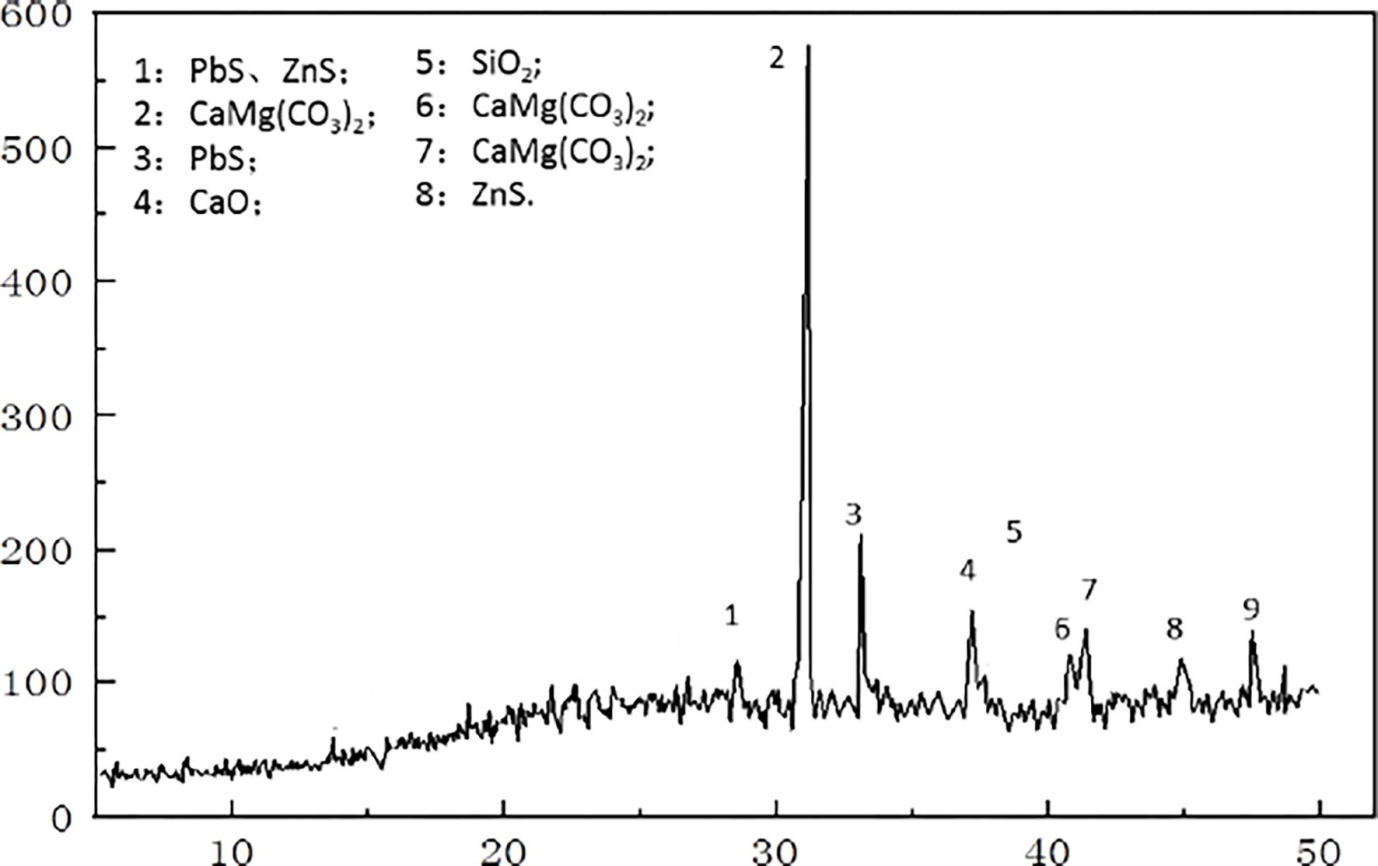
XRD pattern of the Pb‒Zn tailings.

**Fig 3:**
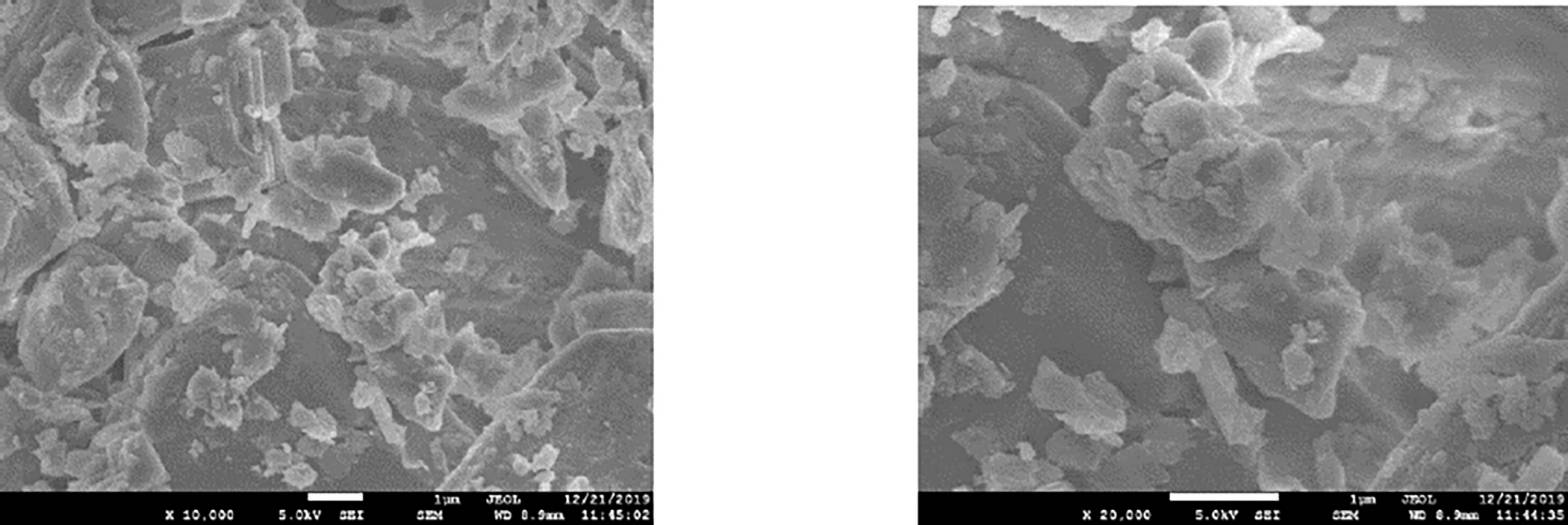
SEM image of the lead-zinc tailings.

The collected tailings samples were air-dried, ground finely, and passed through 200 mesh; one portion of the samples was subjected to XRD, and the other portion was compacted to make block samples. The samples were ground, and reasonably sized samples were taken for scanning electron microscopy (SEM) observation. Care was taken to dry the sample before observation. The sample was glued to a special conductive adhesive and pumped to vacuum after gold spraying treatment. Finally, the samples were observed with different magnification lenses. In this scan, 3 points on the sample were selected for observation, and then 2 groups of images with distinct characteristics were selected as shown in Figs 2 and 3. Analysis of the chemical forms of lead and zinc [33]. There was no difference in the composition of tailings sand samples from multiple collection points in the tailing pond, and the differences in the microscopic properties of tailings at different depths were not studied.

The mineral phases of plagioclase, dolomite, calcite, goethite, gypsum, pyrite, sphalerite, and quartz carbonate in the tailings can be clearly observed in the XRD patterns [34]. Further scanning electron microscopy of the Pb‒Zn tailings sample revealed that the mineral composition was consistent with that observed in the XRD pattern. This confirms the accuracy of this technique, as SEM can also reveal the presence of these materials in the sample. Micrographs of the Pb‒Zn tailings show irregular short flakes, some of which are subject to physical, chemical and biological processes that lead to uneven surface dissolution. This may be the result of long-term weathering of lead-zinc tailings. The surface morphology also shows mineral phases such as quartz sand and sphalerite.

The bulk density, moisture content, pH and organic matter content of the mineral sands of the tailings profile at different depths are shown in Table 2.

**Table 2:** Physical and chemical properties of tailing samples at each depth of profile.

Profile depth (cm)	Bulk density (g·cm^-3^)	Moisture content (%)	Organic matter (%)	pH
0~10	1.27	7.25	2.1	6.5
10~20	1.34	15.47	2.83	6.8
20~30	1.45	21.2	3.86	6.8
30~40	1.18	19.5	3.64	6.8
40~50	1.4	30.85	3.07	6.7
50~60	1.46	31.59	1.79	6.8
60~70	1.24	51.42	2.35	6.7
70~80	1.46	32.96	2.97	6.8
80~90	1.39	31.2	3.28	6.8
90~100	1.18	48.7	2.18	6.7
100~110	1.49	24.62	1.76	6.6
110~120	1.51	28.79	4.38	6.9
120~130	1.46	34.77	1.46	6.9
130~140	1.21	53.64	2.78	6.8
140~150	1.56	11.86	1.37	7.1
150~160	1.56	12.87	3.14	7.0
160~170	1.48	10.61	1.47	7.1
170~180	1.51	14.71	1.1	7.2
180~190	1.56	16.39	1.18	6.9
190~200	1.44	27.15	1.19	6.8

### Spatial distribution characteristics of heavy metals around the tailings area

Soil samples from the industrial plaza and surrounding villages in the Qingshan lead-zinc mining area were measured for heavy and hazardous metals. The total heavy metal concentrations, average values, standard deviations and coefficients of variation are shown in Table 3.

1. The industrial plaza of the lead-zinc mine is approximately 1~5 km away from the tailing pond. Compared with the background values of local soil elements, the mean values of the eight elements measured, Pb, Zn, Cu, Hg, As, Cr, Cd, and Ge, were 5.73, 7.51, 4.44, 1.48, 1.91, 3.27, 1.87, and 0.85 times greater, respectively. The extent of heavy metal contamination of soil in the industrial plaza of the Pb‒Zn mine decreased in the order of Zn > Pb > Cu > Cr > As > Cd > Hg > Ge in terms of the number of exceeding local background values. Except for Ge, which did not exceed the local soil background value, the other seven elements exceeded the local soil background value, with Pb, Zn, Cu and Cr exceeding by the largest amount. This shows that the accumulation of tailings from tailings ponds may have an impact on the heavy metal content of the soil in industrial plazas. According to the standard deviation and coefficient of variation of the heavy metal contents at each sampling site, the coefficients of variation of Pb, Cu and As were greater than 0.1, indicating a high degree of dispersion. To some extent, the levels of these three elements are subject to greater external disturbance factors.
2. The villages around the lead-zinc mine are 5~10 km away from the tailings pond. The mean values of the eight elements Pb, Zn, Cu, Hg, As, Cr, Cd, and Ge measured were 3.64, 2.84, 1.23, 1.24, 2.88, 1.06, 1.43, and 0.83 times greater than the background values of the local soil elements, respectively. The high total amount of Pb, Zn and As among the heavy metals measured in the villages around the mine site indicates that these three heavy metal elements are highly accumulated within the surrounding villages. The contents of all other detected elements were similar in range to the background values. This is mainly attributable to the distance between the villages and the sources of heavy metal pollution from the tailings ponds and the limited extent of contamination of village soils by the action of precipitation and wind and sand. Therefore, the soil around the village has low levels of heavy metals. The results of the standard deviation and coefficient of variation calculations for the heavy metal elements showed a large coefficient of variation for the heavy metal as in village soils. There is a very strong level of anthropogenic disturbance, mainly the result of heavy fertilizer application on village farmlands [35].

**Table 3:** Statistical results of the total heavy metals in the soil around the Qingshan lead-zinc mine.

Project	Sampling location	Pb	Zn		Cu		Hg		As		Cr		Cd		Ge
Total amount of heavy metals /mg·kg^-1^	Industrial Plaza 1	121.43	558.39		70.23		0.094		18.16		174.91		0.21		1.47
	Industrial Plaza 2	155.75	576.31		65.18		0.081		17.75		198.39		0.21		1.52
	Industrial Plaza 3	137.78	504.11		86.92		0.089		13.86		175.25		0.24		1.48
	Village 1	95.27	188.75		22.91		0.077		24.83		55.47		0.17		1.49
	Village 2	80.35	224.92		18.38		0.081		29.87		58.74		0.16		1.51
	Village 3	87.96	205.98		20.27		0.064		20.36		63.6		0.18		1.35
Mean /mg·kg^-1^	Industrial Plaza	138.32	546.27		74.11		0.088		51.35		182.85		0.22		1.49
	Village	87.86	206.55		20.52		0.074		16.33		59.27		0.17		1.45
Standard deviation	Industrial Plaza	14.01	30.69		9.28		0.005		16.59		10.98		0.014		0.02
	Village	6.09	14.77		1.85		0.007		25.02		3.34		0.008		0.07
Coefficient of variation	Industrial Plaza	0.1	0.05		0.12		0.06		0.11		0.06		0.06		0.01
	Village	0.06	0.07		0.09		0.09		0.15		0.05		0.04		0.04
Soil background level /mg·kg^-1^		24.14		72.73		16.69		0.06		8.69		55.92		0.12	1.75

Note: The "background level" in the table refers to the background level of local soil heavy metals.

### Characteristics of the distribution of heavy metal chemical forms around the tailings area

The distribution and morphological characteristics of heavy metals in the soil media around a mine environment are closely related to their transport and transformation [36]. The main chemical forms include the exchangeable state, easily reducible state, easily oxidizable state and residual state. These states can exhibit different environmental behaviours and characteristics. A greater proportion of exchangeable states in the metal form indicates a greater transport capacity in the soil. The readily reducible state, i.e., the oxidized state of Pb‒Zn, may cause decomposition of the oxides and their release for transport when soil environmental conditions change. This has a significant impact on the soil environment. In the easily oxidizable state, i.e., the organic matter-bound state, organic matter in the soil can form complexes with heavy metals. The transport and transformation patterns of heavy metals in soils are affected. The effect on the binding form is not obvious and is "overridden" by other deposition factors [37]. Normally, changes in the pH, intensity and concentration of heavy metal ions in the environment are due to precipitation and dissolution, adsorption and desorption, and oxidation and reduction mechanisms that cause changes in the morphology of heavy metals in the soil.

The solubilization capacity of chemical extractants for heavy elements in different binding states was used to classify the proportion of each state. This led to the analysis of the pattern of heavy metal contamination in the soil.

**Fig 4:**
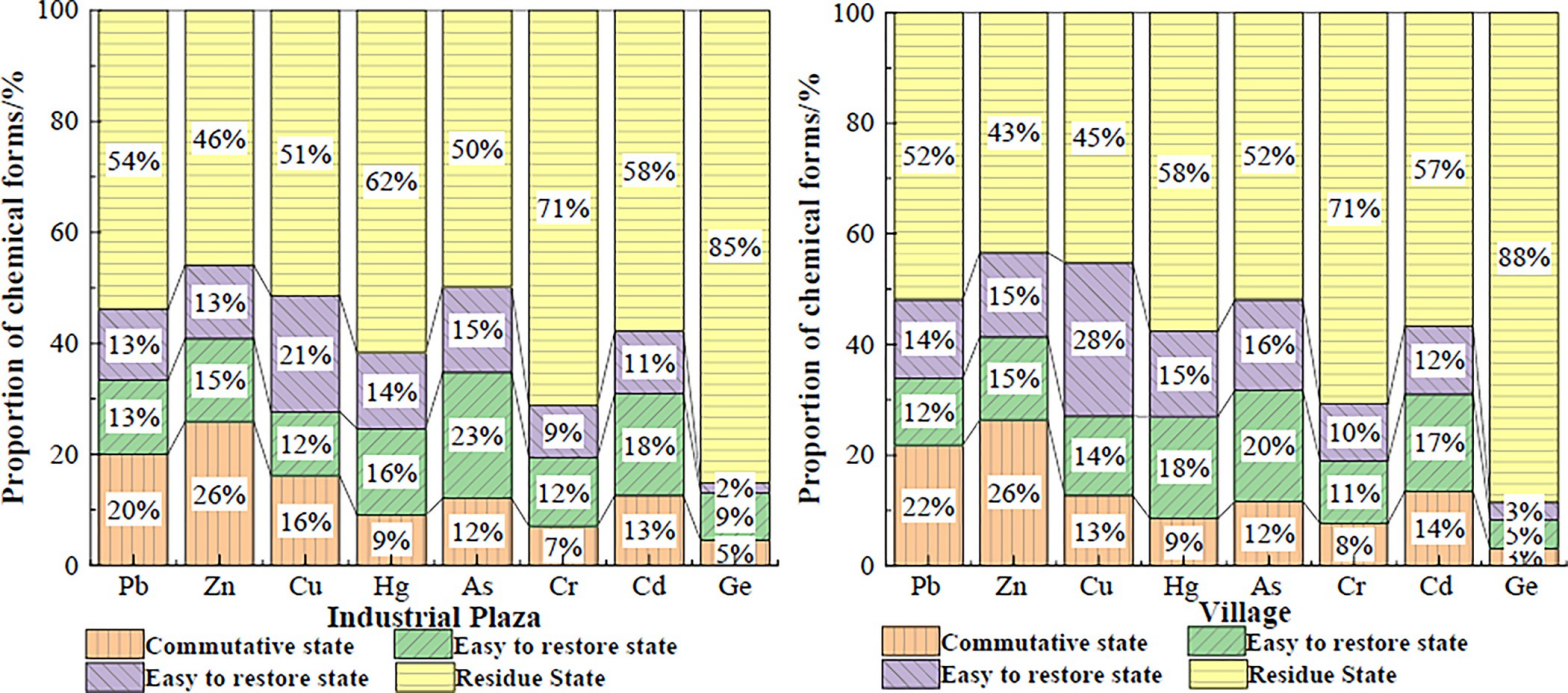
Proportion of chemical forms of heavy metals in soils.

In Fig 4, the proportions of heavy metal chemical forms in the soil around the mine site are visually represented. The industrial plaza and village soils had the highest proportions of exchangeable heavy metals Pb and Zn. This indicates that these two heavy metals have the strongest transport activity in the study area. Presently, the heavy metal ions Pb and Zn are mainly bound to the particle surface by exchange adsorption. The environmental media around the mine site are transported and transformed into natural media, thus affecting the water quality and soil around the mine site. When heavy metals and harmful substances are absorbed by plants and animals, they can cause great harm to biological communities and the environment.

Soils around industrial plazas and villages had the highest contents of exchangeable Pb and Zn and the highest percentage of easily reducible As. The contents of Hg, Cd and Ge in the easily reducible state and the proportion of the easily reducible state in the soils in and around the industrial plaza were low. This indicates that Pb, Zn and As are potentially polluting elements in the soils around industrial plazas and villages. These readily reducible heavy metals are in stronger ion-bonded forms. They will be reduced to an exchangeable state when the redox potential decreases, the pH decreases or oxygen deficiency occurs, causing severe environmental pollution [38]. Therefore, these potential hazards need to be noted.

In summary, the lateral migration of heavy metals can expand the scope of the original contamination. Accurate prediction of heavy metal migration is necessary to control the migration of heavy metals [39]. The stronger the transport capacity of soil heavy metal elements, the more serious the pollution of the surrounding environmental elements. The transport capacity can be expressed as the sum of the percentages of the three forms: exchangeable, oxidizable and reducible. Thus, the order of transport capacity of the eight metals around the industrial plaza and the village are Pb > Zn > As > Cu > Cr > Cd > Hg > Ge and Pb > Zn > Cu > As > Cr > Cd > Hg > Ge. There is some similarity in the distribution of the chemical forms of the eight heavy metals within the industrial plaza and the village surroundings. Cr, Cu, Zn and As are mainly found in the residual state, while Cd and Pb are mainly in the Fe-Mn oxide bound and residual states [40]. The transport capacity of Pb and Zn is greatest in soils around industrial plazas and villages, followed by that of Cu and As. Hg, Cr, Cd, with Ge the most weakly transported at the 2 sites. This indicates that Pb and Zn in the soils around industrial plazas and villages have the greatest impact on the surrounding environment. Heavy metals have the greatest potential ecological impact in the region. Cu and As are strongly active under reducing and oxidizing conditions, and their potential ecological hazards cannot be ignored. Hg had the lowest levels among the heavy metals. The proportions of the first three forms of Ge are basically less than 10%, and its potential ecological hazards can be neglected.

## Prediction model for heavy metal pollution concentrations in the Pb‒Zn tailings area

### Historical monitoring data and its index smoothing calculation

With economic development, soil heavy metal pollution has become a more serious concern. This leads to soil resource degradation and ecosystem deterioration [41]. Exponential smoothing was proposed by Robert G Brown. Robert G Brown, considered the stability or regularity of the dynamics of a time series. The time series can be reasonably extrapolated downstream. Predictions of pollutant concentrations can be inferred from known monitoring data. The exponential smoothing forecasting method is a deterministic smoothing forecasting method. Essentially, exponential smoothing averages are calculated to smooth the time series and eliminate random fluctuations in the historical statistical series in order to identify its major trends. The idea of the exponential smoothing method is to take full account of the indicators of the current cycle and the indicators of the previous cycle. With this approach, better predictions can be obtained [42].

The annual output of the Qingshan Mine is 3.5×10^4^ t of lead and zinc metal, and the total emissions of tailings sand are 30×10^5^ t/a. Except for a small portion of the trailing sand used for downhole filling, the remainder is stockpiled in trailing sand storage. Downstream of the tailings sand reservoir, heavy metals accumulate in farmlands through dust and tailing sand water discharge. To ensure the validity of the data, our team visited the companies responsible for the tailings storage areas and obtained the relevant monitoring data files through thorough organization and review. The heavy metal concentrations in the soil from 2008 to 2016 were investigated and the results shown in Table 4.

**Table 4:** Concentrations of heavy metals in agricultural soils around a tailing pond from 2008–2016 (mg/kg).

Year Element	2008 (1)	2009 (2)	2010 (3)	2011 (4)	2012 (5)	2013 (6)	2014 (7)	2015 (8)	2016 (9)
Pb	167.5	220.0	234.9	224.8	263.2	296.8	268.4	262.0	295.0
Zn	368.9	888.0	2339.0	1521.0	2704.0	1267.0	1152.0	1936.0	1863.0

According to the exponential smoothing method, the assumed future forecast values are related to known past data. The near-term data have a greater impact on the forecasted values, and the forwards data have a smaller impact on the forecasted values [43]. Therefore, this method uses the weighted average of the actual value of the current period and the index smoothing value of the previous period as the index smoothing value of the current period. The mathematical expression of the forecast value for the next period is:

$$S_{t}^{\left(1\right)}={ay}_{t}+(1-a)S_{t-1}^{\left(1\right)}$$ (1)

where $S_{t}^{\left(1\right)}$ is the prediction of *y*_*t*+1_ at moment *t+1* and is denoted as ${ŷ}_{t+1}^{\left(1\right)}$, also known as ${ŷ}_{t+1}^{\left(1\right)}$ is the smoothed prediction of *y*_*t*+1_; *a* is the smoothing factor.

When the time series has a smoothed trend, only Eq (1) needs to be used. Eq (1) is also called primary exponential smoothing. When the time series has a linear trend, it must be estimated by exponential smoothing twice. The nonlinear trend is estimated by 3 exponential smoothing steps. The equations for 2- and 3-fold exponential smoothing are

**Fig 5:**
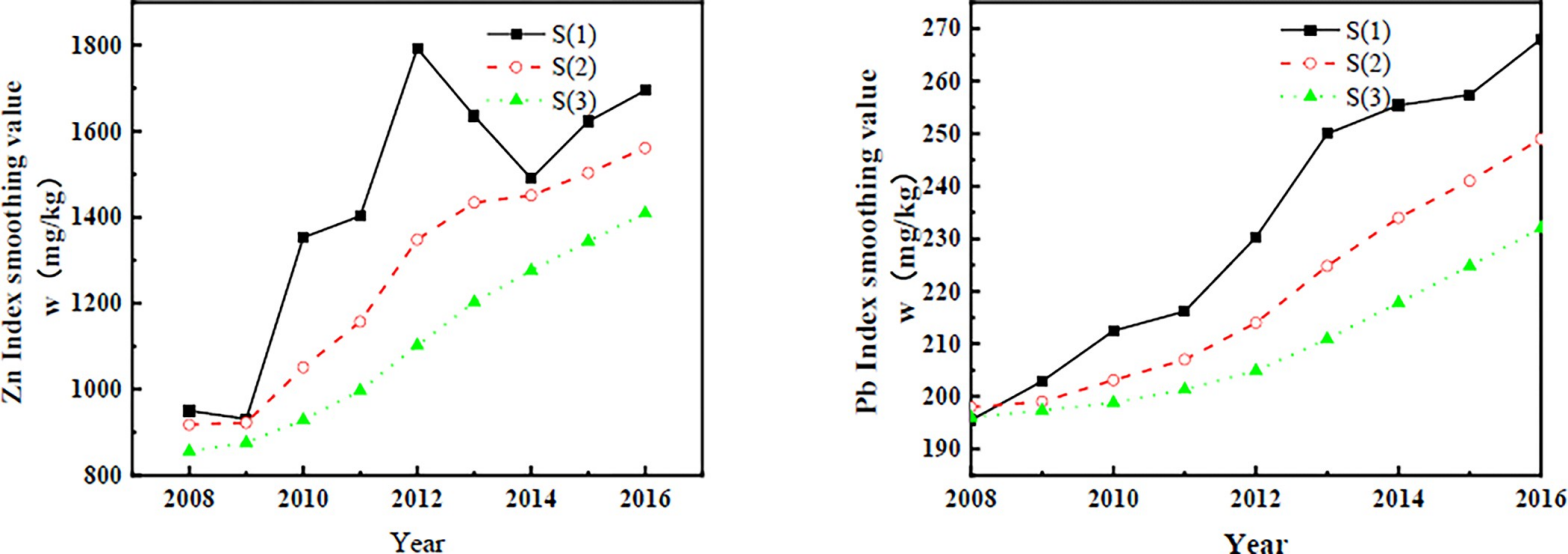
Smoothed number of heavy metal concentration indices in agricultural land near mine tailing dams (2008–2016).

The purpose of exponential smoothing can be calculated by smoothing layer by layer. The influence of random factors is eliminated, and the underlying trend of the forecast is identified. From the time series monitoring data, the time series of this example shows a nonlinear trend. Therefore, the three times exponential smoothing method was used to calculate this series. The fluctuations in the time series data and the principle of selecting smoothing coefficients were considered. If the time series has irregular ups and downs but the long-term trend is close to a stable constant, a smaller value of *a* (taken between 0.05 and 0.2) is necessary. If the time series tends to change rapidly and significantly, *a* should take a larger value (between 0.3 and 0.6). If the time series changes slowly, a smaller value (generally between 0.1 and 0.4) should also be chosen. A smoothing factor of 0.3 is appropriate for this example. The average of the first 3 data points of the sequence is also taken as the initial value. The exponential smoothing values are calculated according to Eqs 1–3, and the calculation results are shown in Fig 5.

### Mathematical modelling of pollution prediction

After calculating the exponential smoothing values one at a time, the corresponding exponential smoothing prediction models can be built for different forms of variations. The soil heavy metal concentration in this mining area has a nonlinear relationship with time, so a nonlinear exponential smoothing prediction model was chosen. The nonlinear model is *a* quadratic equation containing three model parameters:

$$w_{t+L}=a_{t}+b_{t}L+c_{t}L^{2}$$ (4)

where *w*_*t*+*L*_ is the predicted value of the soil heavy metal concentration; *L* is the number of sequences from the current number of sequences *t* to the number of sequences requiring prediction time; and *a*_*t*_, *b*_*t*_, and *c*_*t*_ are parameters.


$$a_{t}={3S}_{t}^{\left(1\right)}-{3S}_{t}^{\left(2\right)}+S_{t}^{\left(3\right)}$$ (5)

$$b_{t}=\frac{a}{{2(1-a)}^{2}}\left[(6-5a)S_{t}^{\left(1\right)}-2(5-4a)S_{t}^{\left(2\right)}+(4-3a)S_{t}^{\left(3\right)}\right]$$ (6)

$$c_{t}=\frac{a^{2}}{{2(2-a)}^{2}}\left[S_{t}^{\left(1\right)}-2S_{t}^{\left(2\right)}+S_{t}^{\left(3\right)}\right]$$ (7)

The starting point of the prediction of the time series is 2016, and the number of series t is 9. The exponential smoothing values in Fig 5 are substituted into Eqs (5)–(7) and calculated as follows:

Pb: *a*_9_ = 289, *b*_9_ = 10.168, *c*_9_ = 0.0312

Zn: *a*_9_ = 1811, *b*_9_ = 40.858, *c*_9_ = -0.281.

*a*_9_, *b*_9_, and *c*_9_ are substituted into Eq (4), the smoothed prediction mathematical model of the heavy metal concentration contamination index of agricultural land around the tailings pond in this mine area.

Pb: *w*_9+*L*_ = 289+10.168 L+0.0312L2;

Zn: *w*_9+*L*_ = 1811+40.858 L-0.281*L*^2^.

### Pollution concentration prediction calculation example and corresponding model accuracy analysis

**Fig 6:**
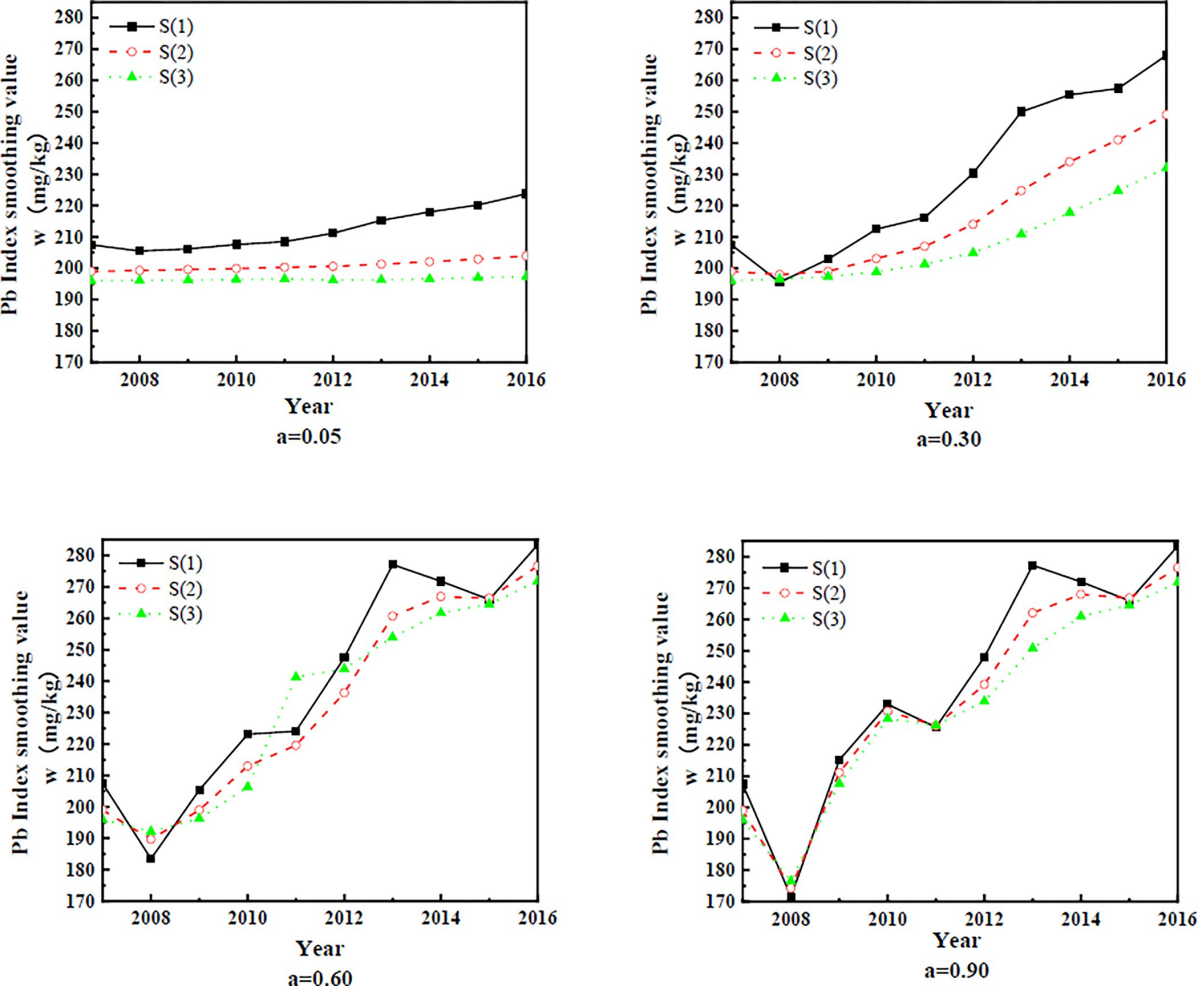
Smoothing values of the soil heavy metal concentration indices at different times.

The whole prediction process demonstrates that the accuracy of the prediction is well-related to the choice of *a*. To validate the above mathematical model, the prediction results are compared for different *a* values. Smoothing coefficients of 0.05, 0.3, 0.6 and 0.9 were used to make post hoc predictions of historical data on lead concentrations in soil. The exponential smoothing values are calculated according to Eqs (1)–(3). The calculation results are shown in Fig 6.

**Fig 7:**
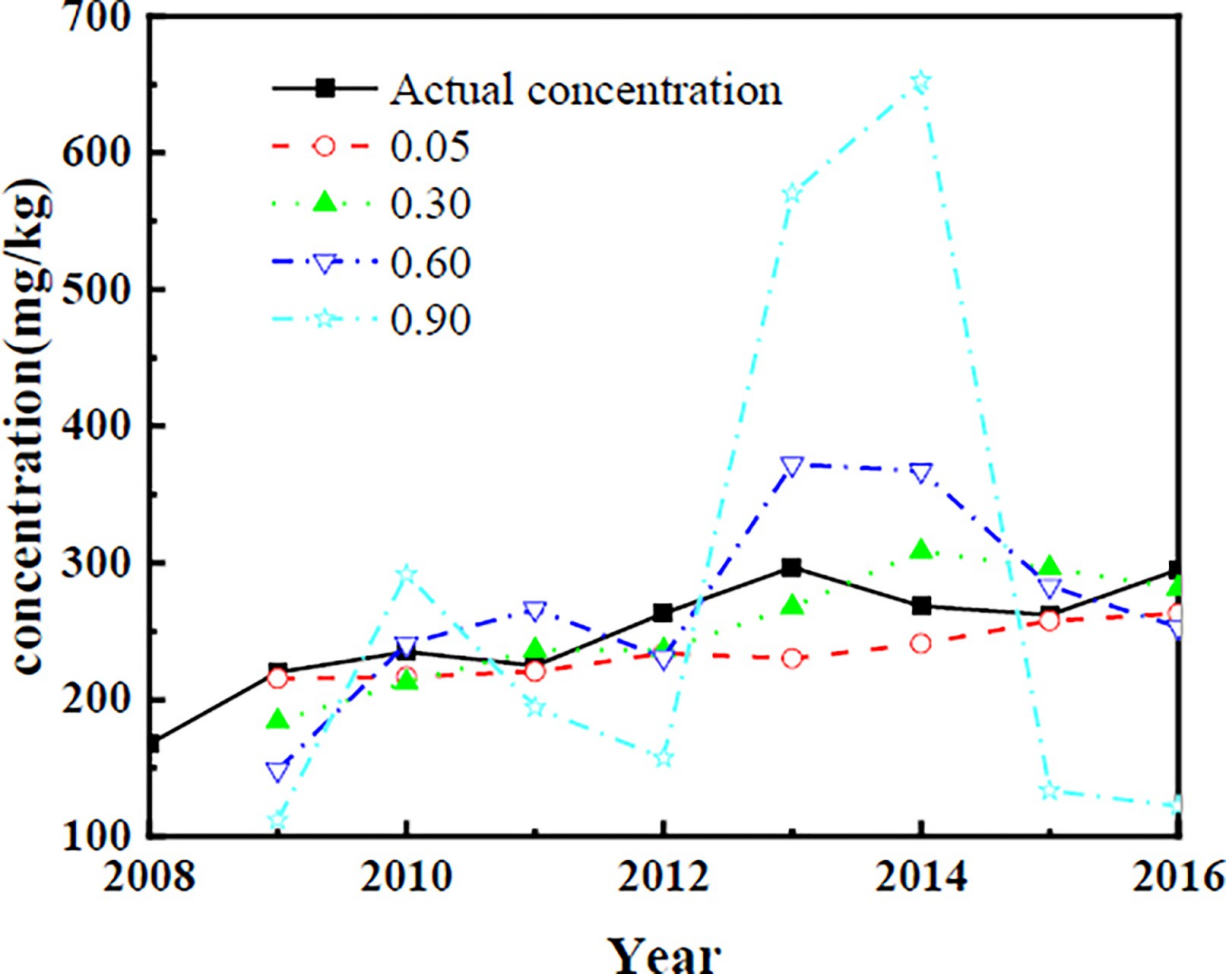
Prediction of soil lead concentration under different smoothing coefficients.

Due to the different starting points of time series forecasting and different mathematical models of forecasting, historical data forecasts are calculated. First, a mathematical model with different *a* values and prediction starting points for each time series is developed according to Fig 6. The results of the predicted soil lead concentration and absolute error calculation are shown in Fig 7.

**Fig 8:**
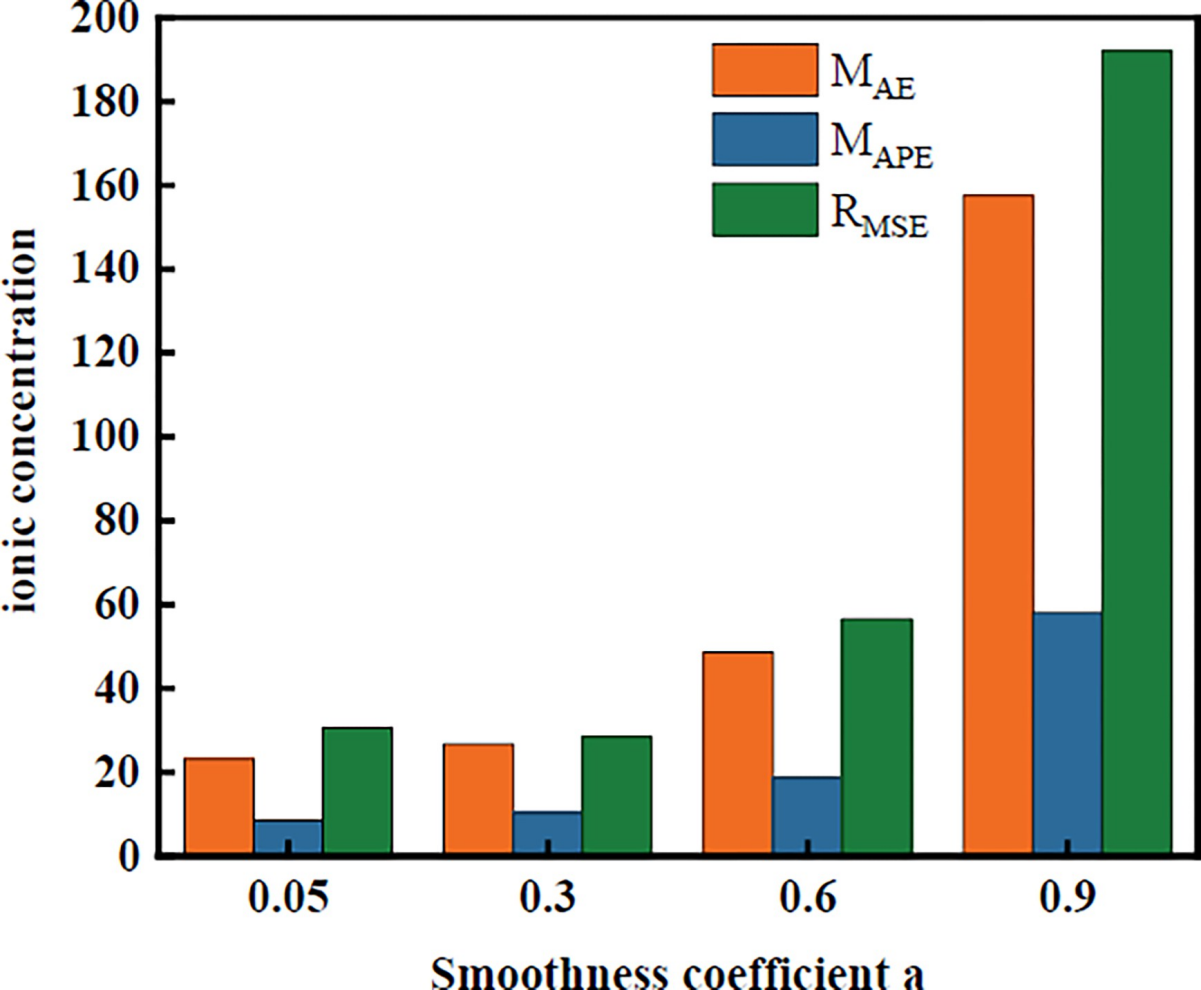
Error analysis of the Pb concentration with different smoothing coefficients.

The absolute mean error (*M*_AE)_, mean absolute error (*M*_APE)_ and mean squared error (*R*_MSE)_ are calculated according to Fig 7, and the results are shown in Fig 8.

From the calculation errors, the average absolute errors were 8.49 and 10.28 when the smoothing coefficients were 0.05 and 0.30, respectively. The derived prediction model can be used as a model for predicting soil heavy metal concentrations around mine tailings ponds. However, according to the analysis of the raw data of each predictor and the principle of the selection of *a*, the measured values of Zn and Pb fluctuated widely in the time series from 2008 to 2016. This may be due to the different spatiotemporal characteristics and contamination mechanisms of the different heavy metal time series concentration variations [44]. This requires the application of different forecasting models for prediction. These methods include causality forecasting and traditional decomposition method forecasting.

Heavy metal pollution is a complex physical and chemical process. Any predictive model has certain flaws [45]. The exponential smoothing forecasting method assumes that there is some underlying pattern in the series of the predicted variable. The sequence of this variable reflects both this basic pattern and contains a stochastic component. The objective is to separate the underlying patterns from the random components by "averaging" the sequence variables. Linear exponential smoothing is intended to make this separation more complete and thus increase the accuracy of the prediction.

Here, based on the above analysis and the principle of smoothing coefficient selection, four smoothing coefficients of 0.05, 0.30, 0.60 and 0.90 are selected for prediction using the same dataset. To reduce the influence of past observations on the prediction, *a* should not be chosen as 0.05. According to the results of the prediction accuracy analysis, a smoothing factor of 0.30 is a reasonable choice for this prediction. The prediction accuracy was 89.72%. This prediction model was used to predict the concentration of heavy metal pollution on land around a mine tailings pond in 2026. The sequence number is *L =* 10 for 2016–2026, and the concentration of heavy metal pollution in the agricultural land around the tailings pond of this mine in 2026 is as follows.

Pb: *w*_9+10_ = 289+10.168×10+0.0312×10^2^ = 393.8 (mg/kg)

Zn: *w*_9+*L*_ = 1811+40.858×10–0.281×10^2^ = 2219.02 (mg/kg)

## Discussion

1. This paper mainly investigates the release and migration patterns of Pb ions and Zn ions in lead-zinc tailings. However, it does not comprehensively consider other inorganic salt pollutants in tailings or the permeation and migration of other pollutants. Future research could further explore the impact of inorganic salt pollutants on tailings pond environments.
2. Exponential smoothing is feasible for predicting environmental pollution. The smoothing coefficient is a key factor in its prediction accuracy. Different smoothing coefficients should be selected according to the trend of different heavy metal ion concentrations. Beneficial to increase the accuracy of forecasts. Long-term monitoring and scientific management of the state of soil and water quality in mining areas. In addition, governments, enterprises and individuals should also recognize the serious threat posed by heavy metal pollutants to ecological environments such as soil and water bodies. Strengthen research on mine heavy metal pollutant removal technology and strongly support mine heavy metal pollution control. In future governance processes. In order to prevent heavy metals from being transported and enriched in large quantities in tailings ponds. Adopting certain techniques in advance to remove heavy metals from tailings before they are discharged into tailings ponds. Reducing the continuous input of heavy metals into the tailing pond from the source reduces the threat of the tailing pond to the surrounding ecological environment.
3. The sustainable utilization of lead-zinc tailings is the most effective solution for remediating tailings pollution. Therefore, in future research, comprehensive utilization methods and technologies for tailings should be considered, thereby reducing the harm of heavy metal ion pollutants and minimizing the financial expenditure associated with the treatment and storage of contaminated lead-zinc tailings.
4. In response to the serious soil pollution caused by heavy metals around the Qingshan lead-zinc mining area, it is suggested that phytoremediation technology be utilized for environmental restoration in contaminated areas. This involves intensifying research on plants with certain abilities to eliminate heavy metals. Additionally, afforestation in the vicinity of the mining area could both improve the local ecological environment and effectively control dust, facilitating the transformation of the Qingshan lead-zinc tailings pond.

## Conclusion

Samples were taken within the perimeter of the tailings pond to analyse the distribution characteristics of metal elements and to predict the situation of heavy metal-contaminated soil. These results show that heavy metal pollution is a dynamic process on long-term and large spatial scales.

1. Heavy metal elements around the industrial plaza and village around the tailings pond all exceeded the local soil elemental background values. The total accumulation of Pb and Zn in the tailings sand of the tailings pond ranged from 936.74 to 1212.61 mg/kg and from 1611.85 to 2191.47 mg/kg, representing the highest proportions.
2. Pb, Cu and As in industrial plaza soils are subject to large external disturbance factors. The large coefficient of variation of As in village soils indicates a large factor of anthropogenic disturbance.
3. Various heavy metal assessment methods indicate that heavy metal pollution in mining areas is primarily dominated by Pb and Zn, accompanied by other heavy metal elements such as Cu and Cd. The ecological hazard level in the mining area is ranked as follows: tailings pond > industrial square > surrounding villages.
4. The concentration prediction model established in this paper offers a more accurate reflection of future development trends and is highly practical. The prediction results show that in 2026, the concentrations of the heavy metals Pb and Zn in the soil around this tailing area will be 1.335 and 1.191 times the predicted starting time. The concentrations of the heavy metals Pb and Zn at the prediction starting point are already 3.34 and 3.02 times the upper limits of the environmental standard (according to the environmental standard for gravelly grey calcium soils).

## Supporting information

S1 File Further details on tailing pond and statistical analyses. Supporting information for the manuscript contains additional information on tailing pond, tables reporting speciation distribution of heavy metals in soils of industrial squares and villages and Smoothed values w of heavy metal concentration index in farmland soil around Qingshan tailings pond from 2008 to 2016 (mg/kg). Pb content prediction and error, Exponential smooth number of soil heavy metal concentration with different coefficients a.
(DOCX)

## References

[1] Ngole-Jeme VM, Fantke P. Ecological and human health risks associated with abandoned gold mine tailings contaminated soil. PLoS One. 2017;12(2):e0172517. doi: 10.1371/journal.pone.0172517 . 28222184 PMC5319768

[2] Du C, Niu B, Yi F, Jiang X, Liang L. Impact of inundation range of overtopping dam break of tailings pond under actual terrain conditions. PLoS One. 2023;18(12):e0295056. doi: 10.1371/journal.pone.0295056 . 38055754 PMC10699616

[3] Ke W, Zeng J, Zhu F, Luo X, Feng J, He J, et al. Geochemical partitioning and spatial distribution of heavy metals in soils contaminated by lead smelting. Environ Pollut. 2022; 307:119486. doi: 10.1016/j.envpol.2022.119486 . 35595002

[4] Zeng J, Li C, Wang J, Tang L, Wu C, Xue S. Pollution simulation and remediation strategy of a zinc smelting site based on multi-source information. J Hazard Mater. 2022;433:128774. doi: 10.1016/j.jhazmat.2022.128774 . 35397337

[5] Niu H, Sun Q, Bu Y, et al. Study of the microstructure and oxidation characteristics of residual coal in deep mines. Journal of Cleaner Production. 2022; 373: 133923. 10.1016/j.jclepro.2022.133923.

[6] Hu J, Zhou S, Wu P, Qu K. Assessment of the distribution, bioavailability and ecological risks of heavy metals in the lake water and surface sediments of the Caohai plateau wetland, China. PLoS One. 2017;12(12):e0189295. doi: 10.1371/journal.pone.0189295 . 29253896 PMC5734908

[7] Li W, Zuo Y, Wang L, Wan X, Yang J, Liang T, et al. Abundance, spatial variation, and sources of rare earth elements in soils around ion-adsorbed rare earth mining areas. Environ Pollut. 2022;313:120099. doi: 10.1016/j.envpol.2022.120099 . 36084740

[8] Liu S, Yuan R, Wang X, Yan Z. Soil tungsten contamination and health risk assessment of an abandoned tungsten mine site. Sci Total Environ. 2022;852:158461. doi: 10.1016/j.scitotenv.2022.158461 . 36063943

[9] Fan M, Lin Y, Huo H, Liu Y, Zhao L, Wang E, et al. Microbial communities in riparian soils of a settling pond for mine drainage treatment. Water Res. 2016;96:198–207. doi: 10.1016/j.watres.2016.03.061 . 27055175

[10] Fashola MO, Ngole-Jeme VM, Babalola OO. Heavy Metal Pollution from Gold Mines: Environmental Effects and Bacterial Strategies for Resistance. Int J Environ Res Public Health. 2016;13(11):1047. doi: 10.3390/ijerph13111047 . 27792205 PMC5129257

[11] Wu B, Wang G, Wu J, Fu Q, Liu C. Sources of heavy metals in surface sediments and an ecological risk assessment from two adjacent Plateau reservoirs. PLoS One. 2014;9(7):e102101. doi: 10.1371/journal.pone.0102101 . 25010771 PMC4092113

[12] Liu J, Liu R, Zhang Z, Cai Y, Zhang L. A Bayesian Network-based risk dynamic simulation model for accidental water pollution discharge of mine tailings ponds at watershed-scale. J Environ Manage. 2019;246:821–831. doi: 10.1016/j.jenvman.2019.06.060 . 31228695

[13] Guo Y, Huang P, Zhang W, et al. Leaching of heavy metals from Dexing copper mine tailings pond. Transactions of Nonferrous Metals Society of China, 2013; 23(10): 3068–3075. 10.1016/S1003-6326(13)62835-6.

[14] MacDonald DD, Ingersoll CG, Berger TA. Development and evaluation of consensus-based sediment quality guidelines for freshwater ecosystems. Arch Environ Contam Toxicol. 2000;39(1):20–31. doi: 10.1007/s002440010075 . 10790498

[15] Sarwar N, Imran M, Shaheen MR, Ishaque W, Kamran MA, Matloob A, et al. Phytoremediation strategies for soils contaminated with heavy metals: Modifications and future perspectives. Chemosphere. 2017;171:710–721. doi: 10.1016/j.chemosphere.2016.12.116 . 28061428

[16] Yao Y, Tong L, Zhao R, Wang Q, Qiu J, Wang F, et al. Leaching of heavy metal(loid)s from historical Pb-Zn mining tailing in abandoned tailing deposit: Up-flow column and batch tests. J Environ Manage. 2023;325(Pt A):116572. doi: 10.1016/j.jenvman.2022.116572 . 36419286

[17] Liu B, Sun H, Peng T, et al. Transport and transformation of uranium and heavy metals from uranium tailings under simulated rain at different pH. Environmental Chemistry Letters, 2020;18: 495–503. 10.1007/s10311-019-00951-4.

[18] Zhang J, Xu Y, Wu Y, et al. Dynamic characteristics of heavy metal accumulation in the farmland soil over Xiaoqinling gold-mining region, Shaanxi, China. Environmental earth sciences, 2019;78: 1–11. 10.1007/s12665-018-8013-2.

[19] Carvalho F P, Oliveira J M, Malta M, et al. Radioanalytical assessment of environmental contamination around non-remediated uranium mining legacy site and radium mobility[J]. Journal of Radioanalytical and Nuclear Chemistry, 2014; 299: 119–125. 10.1007/s10967-013-2734-1.

[20] Kushwaha A, Hans N, Kumar S, Rani R. A critical review on speciation, mobilization and toxicity of lead in soil-microbe-plant system and bioremediation strategies. Ecotoxicol Environ Saf. 2018;147:1035–1045. doi: 10.1016/j.ecoenv.2017.09.049 . 29976006

[21] Tang B, Xu H, Song F, Ge H, Yue S. Effects of heavy metals on microorganisms and enzymes in soils of lead-zinc tailing ponds. Environ Res. 2022;207:112174. doi: 10.1016/j.envres.2021.112174 . 34637758

[22] Liu J, Wang J, Li H, et al. Surface sediment contamination by uranium mining/milling activities in South China. CLEAN–Soil, Air, Water, 2015;43(3): 414–420. 10.1002/clen.201300297.

[23] He Y, Li BB, Zhang KN, Li Z, Chen YG, Ye WM. Experimental and numerical study on heavy metal contaminant migration and retention behavior of engineered barrier in tailings pond. Environ Pollut. 2019;252:1010–1018. doi: 10.1016/j.envpol.2019.06.072 . 31252097

[24] Tabelin CB, Uyama A, Tomiyama S, Villacorte-Tabelin M, Phengsaart T, Silwamba M, et al. Geochemical audit of a historical tailings storage facility in Japan: Acid mine drainage formation, zinc migration and mitigation strategies. J Hazard Mater. 2022;438:129453. doi: 10.1016/j.jhazmat.2022.129453 . 35797786

[25] Dreisinger D, Abed N. A fundamental study of the reductive leaching of chalcopyrite using metallic iron part I: kinetic analysis. Hydrometallurgy, 2002; 66(1–3): 37–57. 10.1016/S0304-386X(02)00079-8.

[26] Georgiou D, Papangelakis V G. Sulphuric acid pressure leaching of a limonitic laterite: chemistry and kinetics. Hydrometallurgy, 1998; 49(1–2): 23–46. 10.1016/S0304-386X(98)00023-1.

[27] Liu Z, Fei Y, Shi H, Mo L, Qi J. Prediction of high-risk areas of soil heavy metal pollution with multiple factors on a large scale in industrial agglomeration areas. Sci Total Environ. 2022;808:151874. doi: 10.1016/j.scitotenv.2021.151874 . 34826472

[28] Xie Y, Chen TB, Lei M, Yang J, Guo QJ, Song B, et al. Spatial distribution of soil heavy metal pollution estimated by different interpolation methods: accuracy and uncertainty analysis. Chemosphere. 2011;82(3):468–76. doi: 10.1016/j.chemosphere.2010.09.053 . 20970158

[29] Dong J, Dai W, Xu J, Li S. Spectral Estimation Model Construction of Heavy Metals in Mining Reclamation Areas. Int J Environ Res Public Health. 2016;13(7):640. doi: 10.3390/ijerph13070640 . 27367708 PMC4962181

[30] Zhang S, Shen Q, Nie C, Huang Y, Wang J, Hu Q, et al. Hyperspectral inversion of heavy metal content in reclaimed soil from a mining wasteland based on different spectral transformation and modeling methods. Spectrochim Acta A Mol Biomol Spectrosc. 2019;211:393–400. doi: 10.1016/j.saa.2018.12.032 . 30594866

[31] Callesen I., Keck H. & Andersen T.J. Particle size distribution in soils and marine sediments by laser diffraction using Malvern Mastersizer 2000—method uncertainty including the effect of hydrogen peroxide pretreatment. J Soils Sediments 18, 2500–2510 (2018). 10.1007/s11368-018-1965-8.

[32] Khadhar S, Sdiri A, Chekirben A, Azouzi R, Charef A. Integration of sequential extraction, chemical analysis and statistical tools for the availability risk assessment of heavy metals in sludge amended soils. Environmental Pollution, 2020;263: 114543. 10.1016/j.envpol.2020.114543.

[33] Bevandić S, Blannin R, Escobar A G, Bachmann K, Frenzel M, Pinto A, et al. Metal deportment in Pb-Zn mine wastes from a historic tailings pond, Plombières, East Belgium. Minerals Engineering, 2022; 184: 107628. 10.1016/j.mineng.2022.107628.

[34] Chen Q, Luo K, Wang Y, Li X, Zhang Q, Liu Y. In-situ stabilization/solidification of lead/zinc mine tailings by cemented paste backfill modified with low-carbon bentonite alternative. Journal of Materials Research and Technology, 2022; 17: 1200–1210. 10.1016/j.jmrt.2022.01.099.

[35] Feng YX, Yu XZ, Zhang H. A modelling study of a buffer zone in abating heavy metal contamination from a gold mine of Hainan Province in nearby agricultural area. J Environ Manage. 2021;287:112299. doi: 10.1016/j.jenvman.2021.112299 . 33714040

[36] Nemati K, Abu Bakar NK, Abas MR, Sobhanzadeh E. Speciation of heavy metals by modified BCR sequential extraction procedure in different depths of sediments from Sungai Buloh, Selangor, Malaysia. J Hazard Mater. 2011;192(1):402–10. doi: 10.1016/j.jhazmat.2011.05.039 . 21684080

[37] Nabuyanda MM, Kelderman P, Sankura MG, Rousseau D, Irvine K. Investigating the effect of Eh and pH on binding forms of Co, Cu, and Pb in wetland sediments from Zambia. J Environ Manage. 2022;319:115543. doi: 10.1016/j.jenvman.2022.115543 . 35820307

[38] Yang J, Zhang X, Chen M, Huang Y, Tian B, Wang N, et al. Versatile hydrogen-bonded organic framework (HOF) platform for simultaneous detection and efficient removal of heavy metal ions. Journal of Environmental Chemical Engineering, 2022; 10(6): 108983. 10.1016/j.jece.2022.108983.

[39] Qiao P, Wang S, Li J, Zhao Q, Wei Y, Lei M, et al. Process, influencing factors, and simulation of the lateral transport of heavy metals in surface runoff in a mining area driven by rainfall: A review. Sci Total Environ. 2023;857(Pt 1):159119. doi: 10.1016/j.scitotenv.2022.159119 . 36183764

[40] Zhang YR, Che FF, Fu ZH, Xu Y, Li W. Distribution and Potential Ecological Risk Assessment of Heavy Metals in Sediments of Lake Qinghai. Huan Jing Ke Xue. 2022;43(6):3037–3047. Chinese. doi: 10.13227/j.hjkx.202108201 . 35686773

[41] Wu Y, Li X, Yu L, Wang T, Wang J, Liu T. Review of soil heavy metal pollution in China: Spatial distribution, primary sources, and remediation alternatives. Resources, Conservation and Recycling, 2022; 181: 106261. 10.1016/j.resconrec.2022.106261.

[42] Billah B, King M L, Snyder R D, et al. Exponential smoothing model selection for forecasting. International journal of forecasting, 2006, 22(2): 239–247. 10.1016/j.ijforecast.2005.08.002.

[43] Guoli L, Yinda Z, Chao W. Forecast models of heavy metal contamination near tailing dam and their application. J. Cent. South Univ, 2004; 1009–1013. doi: 10.1111/j.1365-2044.2007.05102.x

[44] Yang L, Meng F, Ma C, Hou D. Elucidating the spatial determinants of heavy metals pollution in different agricultural soils using geographically weighted regression. Sci Total Environ. 2022;853:158628. doi: 10.1016/j.scitotenv.2022.158628 . 36087662

[45] Chen X, Ding Z T, Khan A, Kakade A, Ye Z, Li R, et al. Current status and development of remediation for heavy metals in China. Applied Environmental Biotechnology, 2019; 4(2): 5–18. 10.26789/AEB.2019.01.005.

