# Supplementary material for: Prediction of heavy metal ion distribution and Pb and Zn ion concentrations in the tailing pond area
Source: PLoS One. 2024 Sep 26;19(9):e0308916. doi: 10.1371/journal.pone.0308916 (PMC11426534; doi:10.1371/journal.pone.0308916)
Supplement: S1 File — Supporting information for the manuscript contains additional information on tailing pond, tables reporting speciation distribution of heavy metals in soils of industrial squares and villages and Smoothed values w of heavy metal concentration index in farmland soil around Qingshan tailings pond from 2008 to 2016 (mg/kg). Pb content prediction and error, Exponential smooth number of soil heavy metal concentration with different coefficients a. (DOCX) [file pone.0308916.s001.docx]

**Table 1. Speciation distribution of heavy metals in soils of industrial squares and villages.**

| Tailing pond area | Heavy metal | Content（mg⋅kg^-1^） | | | | Percentage of（%） | | | |
| --- | --- | --- | --- | --- | --- | --- | --- | --- | --- |
|  |  | Exchangeable state | Easily reducible state | Easily oxidizable state | Residual state | Exchangeable state | Easily reducible state | Easily oxidizable state | Residual state |
| Industrial Plaza | Pb | 27.761 | 18.410 | 17.594 | 74.554 | 20.07 | 13.31 | 12.72 | 53.9 |
|  | Zn | 141.320 | 81.777 | 72.272 | 250.902 | 25.87 | 14.97 | 13.23 | 45.93 |
|  | Cu | 11.969 | 8.560 | 15.445 | 38.137 | 16.15 | 11.55 | 20.84 | 51.46 |
|  | Hg | 0.008 | 0.014 | 0.012 | 0.054 | 9.09 | 15.54 | 13.68 | 61.69 |
|  | As | 6.198 | 11.651 | 7.923 | 25.577 | 12.07 | 22.69 | 15.43 | 49.81 |
|  | Cr | 12.946 | 22.545 | 17.078 | 130.281 | 7.08 | 12.33 | 9.34 | 71.25 |
|  | Cd | 0.028 | 0.040 | 0.025 | 0.127 | 12.58 | 18.32 | 11.32 | 57.78 |
|  | Ge | 0.066 | 0.124 | 0.026 | 1.235 | 4.52 | 8.53 | 1.81 | 85.14 |
| Village | Pb | 19.136 | 10.570 | 12.511 | 45.643 | 21.78 | 12.03 | 14.24 | 51.95 |
|  | Zn | 54.426 | 31.065 | 31.437 | 89.622 | 26.35 | 15.04 | 15.22 | 43.39 |
|  | Cu | 2.612 | 2.938 | 5.690 | 9.279 | 12.73 | 14.32 | 27.73 | 45.22 |
|  | Hg | 0.006 | 0.014 | 0.011 | 0.043 | 8.58 | 18.39 | 15.46 | 57.57 |
|  | As | 1.901 | 3.282 | 2.663 | 8.483 | 11.64 | 20.1 | 16.31 | 51.95 |
|  | Cr | 4.534 | 6.721 | 6.075 | 41.939 | 7.65 | 11.34 | 10.25 | 70.76 |
|  | Cd | 0.023 | 0.030 | 0.021 | 0.096 | 13.52 | 17.49 | 12.37 | 56.62 |
|  | Ge | 0.046 | 0.075 | 0.047 | 1.283 | 3.16 | 5.14 | 3.23 | 88.47 |

Table 1 shows that the distribution and morphological characteristics of heavy metals in the soil medium around the mining area are closely related to their migration and transformation. The main chemical forms include exchangeable state, easily reduced state, easily oxidized state and residual state. These states may show different environmental behaviors and characteristics. The reducible state refers to the oxidation state of lead and zinc. When the soil environmental conditions change, it may lead to the decomposition of oxides, thus releasing and transporting, causing serious damage to the soil ecosystem. The easily oxidized state indicates that organic matter and heavy metal elements form complexes, and the organic matter in the soil can affect the migration and transformation of heavy metals in the soil. Generally, the changes of pH, ionic strength and concentration in the environmental system affect the morphological changes and environmental behavior of heavy metals through precipitation and dissolution, adsorption and desorption, oxidation and reduction. The dissolution ability of chemical extractant to different bound heavy metal elements was used to divide the proportion of each state, so as to analyze the form of heavy metal pollution in soil.

**Table 2. Smoothed values *w* of heavy metal concentration index in farmland soil around Qingshan tailings pond from 2008 to 2016 (mg/kg).**

| Sequence ( year ) | Pb | | | Zn | | |
| --- | --- | --- | --- | --- | --- | --- |
|  | *S*^(1)^ | *S*^(2)^ | *S*^(3)^ | *S*^(1)^ | *S*^(2)^ | *S*^(3)^ |
|  | 207.5 | 199.0 | 196.0 | 1199.0 | 904.0 | 829.0 |
| 1（2008） | 195.5 | 198.0 | 196.0 | 950.0 | 917.8 | 855.6 |
| 2（2009） | 202.9 | 199.0 | 197.3 | 931.0 | 922.0 | 875.5 |
| 3（2010） | 212.5 | 203.1 | 198.8 | 1353.0 | 1051.0 | 928.5 |
| 4（2011） | 216.2 | 207.0 | 201.3 | 1403.0 | 1157.0 | 997.0 |
| 5（2012） | 230.3 | 214.0 | 204.9 | 1793.0 | 1348.0 | 1102.0 |
| 6（2013） | 250.0 | 224.8 | 210.9 | 1635.0 | 1434.0 | 1202.0 |
| 7（2014） | 255.4 | 234.0 | 217.8 | 1490.0 | 1451.0 | 1276.0 |
| 8（2015） | 257.4 | 241.0 | 224.8 | 1623.0 | 1503.0 | 1344.0 |
| 9（2016） | 268.0 | 249.0 | 232.0 | 1695.0 | 1561.0 | 1409.0 |

Table 2 shows the Smoothed values w of heavy metal concentration index in farmland soil around Qingshan tailings pond from 2008 to 2016 (mg/kg). The trend of concentration changes nonlinearly. Therefore, the quadratic exponential smoothing cannot meet the prediction requirements, and the sequence needs to be calculated by the cubic exponential smoothing method.

**Table 3. Exponential smooth number of soil heavy metal concentration with different coefficients a.**

| Sequence ( year ) | *a*=0.05 | | | *a*=0.30 | | | *a*=0.60 | | | *a*=0.90 | | |
| --- | --- | --- | --- | --- | --- | --- | --- | --- | --- | --- | --- | --- |
|  | *S*^(1)^ | *S*^(2)^ | *S*^(3)^ | *S*^(1)^ | *S*^(2)^ | *S*^(3)^ | *S*^(1)^ | *S*^(2)^ | *S*^(3)^ | *S*^(1)^ | *S*^(2)^ | *S*^(3)^ |
|  | 207.5 | 199.0 | 196.0 | 207.5 | 199.0 | 196.0 | 207.5 | 199.0 | 196.0 | 207.5 | 199.0 | 196.0 |
| 1、2008 | 205.5 | 199.3 | 196.1 | 195.5 | 198.0 | 196.6 | 183.5 | 189.7 | 192.2 | 171.5 | 174.3 | 176.5 |
| 2、2009 | 206.2 | 199.6 | 196.2 | 202.9 | 199.0 | 197.3 | 205.4 | 199.0 | 196.3 | 215.2 | 211.1 | 207.6 |
| 3、2010 | 207.6 | 199.9 | 196.4 | 212.5 | 203.1 | 198.8 | 223.1 | 213.0 | 206.3 | 232.9 | 230.7 | 228.3 |
| 4、2011 | 208.5 | 200.3 | 196.6 | 216.2 | 207.0 | 201.3 | 224.1 | 219.6 | 241.3 | 225.6 | 226.1 | 226.2 |
| 5、2012 | 211.2 | 200.6 | 196.2 | 230.3 | 214.0 | 204.9 | 247.5 | 236.3 | 243.9 | 248.0 | 239.2 | 233.9 |
| 6、2013 | 215.3 | 201.3 | 196.3 | 250.0 | 224.8 | 210.9 | 277.1 | 260.7 | 254.0 | 277.3 | 262.1 | 250.8 |
| 7、2014 | 218.0 | 202.1 | 196.6 | 255.4 | 234.0 | 217.8 | 271.8 | 266.9 | 261.7 | 272.0 | 268.0 | 261.0 |
| 8、2015 | 220.2 | 202.9 | 197.0 | 257.4 | 241.0 | 224.8 | 266.0 | 266.4 | 264.5 | 266.0 | 266.8 | 264.5 |
| 9、2016 | 223.8 | 203.9 | 197.3 | 268.0 | 249.0 | 232.0 | 283.4 | 276.6 | 271.8 | 283.4 | 276.5 | 271.8 |

In order to verify the mathematical model in the manuscript, the prediction results of different a were compared. The smoothing coefficients were taken as 0.05, 0.3, 0.6 and 0.9 for post-hoc prediction of the historical data of lead concentration in soil, and the calculated results are shown in Table 3.

**Table 4. Pb concentration forecast and error calculation of farmland soil with different smooth coefficients.**

| Year | Actual concentrations | *a*=0.05 | | *a*=0.30 | | *a*=0.60 | | *a*=0.90 | |
| --- | --- | --- | --- | --- | --- | --- | --- | --- | --- |
|  |  | Predicted value | Error magnitude | Predicted value | Error magnitude | Predicted value | Error magnitude | Predicted value | Error magnitude |
| 2008 | 167.5 |  |  |  |  |  |  |  |  |
| 2009 | 220.0 | 215.3 | 4.7 | 184.2 | 36.8 | 148.6 | 71.4 | 111.8 | 108.2 |
| 2010 | 234.9 | 216.7 | 18.2 | 212.6 | 22.3 | 240.8 | 5.9 | 291.5 | 56.6 |
| 2011 | 224.8 | 220.4 | 4.4 | 236.1 | 11.3 | 266.2 | 41.4 | 194.3 | 30.5 |
| 2012 | 263.2 | 234.0 | 29.2 | 236.0 | 27.2 | 230.7 | 32.5 | 157.3 | 105.9 |
| 2013 | 296.8 | 230.0 | 66.8 | 267.8 | 29.0 | 372.1 | 75.3 | 570.2 | 273.4 |
| 2014 | 268.4 | 241.0 | 27.4 | 308.3 | 39.0 | 367.4 | 99 | 652.8 | 384.4 |
| 2015 | 262.0 | 257.6 | 4.4 | 296.3 | 34.3 | 283.0 | 21 | 133.5 | 128.5 |
| 2016 | 295.0 | 263.0 | 32.0 | 281.3 | 13.7 | 253.0 | 42.0 | 122.0 | 173.0 |

Since the starting point of time series prediction is different, the prediction mathematical model is also different, so to calculate the predicted value of historical data, firstly, we need to establish the mathematical model with different a-values and the starting point of prediction of each time series according to Table 3. After calculating the soil lead concentration prediction and absolute error calculation results are shown in Table 4.

**Table 5. Error analysis of Pb concentration with different smooth coefficients.**

| Error | *a*=0.05 | *a*=0.30 | *a*=0.60 | *a*=0.90 |
| --- | --- | --- | --- | --- |
| *M*_APE_ | 8.49 | 10.28 | 18.6 | 58.1 |
| *R*_MSE_ | 30.6 | 28.38 | 56.55 | 192.0 |

According to Table 4 and then calculate its mean absolute error *M*_APE_ and mean square error *R*_MSE_ respectively, the calculation results are shown in Table 5
